# (6*E*,10*E*) Isopolycerasoidol and (6*E*,10*E*) Isopolycerasoidol Methyl Ester, Prenylated Benzopyran Derivatives from *Pseuduvaria monticola* Induce Mitochondrial-Mediated Apoptosis in Human Breast Adenocarcinoma Cells

**DOI:** 10.1371/journal.pone.0126126

**Published:** 2015-05-06

**Authors:** Hairin Taha, Chung Yeng Looi, Aditya Arya, Won Fen Wong, Lee Fah Yap, Mohadeseh Hasanpourghadi, Mustafa A. Mohd, Ian C Paterson, Hapipah Mohd Ali

**Affiliations:** 1 Department of Chemistry, Faculty of Science, University of Malaya, Kuala Lumpur, Malaysia; 2 Department of Pharmacology, Faculty of Medicine, University of Malaya, Kuala Lumpur, Malaysia; 3 Department of Pharmacy, Faculty of Medicine, University of Malaya, Kuala Lumpur, Malaysia; 4 Department of Medical Microbiology, Faculty of Medicine, University of Malaya, Kuala Lumpur, Malaysia; 5 Department of Oral Biology and Biomedical Sciences, and Oral Cancer Research and Coordinating Centre (OCRCC),Faculty of Dentistry, University of Malaya, Kuala Lumpur, Malaysia; Wayne State University, UNITED STATES

## Abstract

Phytochemicals from *Pseuduvaria* species have been reported to display a wide range of biological activities. In the present study, a known benzopyran derivative, (6*E*,10*E*) isopolycerasoidol (**1**), and a new benzopyran derivative, (6*E*,10*E*) isopolycerasoidol methyl ester (**2**), were isolated from a methanol extract of *Pseuduvaria monticola* leaves. The structures of the isolated compounds were elucidated by spectroscopic methods including 1D and 2D NMR, IR, UV, and LCMS-QTOF, and by comparison with previously published data. The anti-proliferative and cytotoxic effects of these compounds on human breast cancer cell-lines (MCF-7 and MDA-MB-231) and a human normal breast epithelial cell line (MCF-10A) were investigated. MTT results revealed both (**1**) and (**2**) were efficient in reducing cell viability of breast cancer cells. Flow cytometry analysis demonstrated that (**1**) and (**2**) induced cell death via apoptosis, as demonstrated by an increase in phosphotidylserine exposure. Both compounds elevated ROS production, leading to reduced mitochondrial membrane potential and increased plasma membrane permeability in breast cancer cells. These effects occurred concomitantly with a dose-dependent activation of caspase 3/7 and 9, a down-regulation of the anti-apoptotic gene *BCL2* and the accumulation of p38 MAPK in the nucleus. Taken together, our data demonstrate that (**1**) and (**2**) induce intrinsic mitochondrial-mediated apoptosis in human breast cancer cells, which provides the first pharmacological evidence for their future development as anticancer agents.

## Introduction

Many active phytochemicals (glycosides, flavonoids, phenols, steroids, alkaloids and terpenoids) have been shown to exhibit a variety of biological properties [1, 2]. The search for new anticancer agents from natural resources is an active area of research synthetic anticancer drugs such as doxorubicin and taxols are associated with serious side effects [3]. The genus *Pseuduvaria* is a montane forest plant species which belongs to the family Annonaceae. Plants in this genus are in the major group of flowering plants (Angiosperms) that are made up of shrubs and trees usually found in the rainforest population [4]. *Pseuduvaria* species are commonly found in Malaysia, Thailand, Burma, Indonesia and in the north eastern part of Queensland, Australia. There are more than 50 classified and documented *Pseuduvaria* species but only a few have been investigated phytochemically and pharmacologically [5].

A number of *Pseuduvaria* species have been used traditionally for treating cough, fever and stomach ailments. In the Malay Peninsula, the root of *Pseuduvaria setosa* is used to cure cough and relieve fever. The roots are also consumed as a mixture eaten with betel as an aphrodisiac and the fruits are consumed by fruit bats as one of their main diets during the fruiting season [6]. Previous studies have identified isoquinoline alkaloids from *Pseuduvaria* species with interesting pharmacological properties such as cytotoxicity, antituberculosis and antimalarial activities, whereas ethyl acetate extracts of *P*. *macrophylla* exhibited broad spectrum antibacterial properties [7–9].

*P*. *monticola* is a mountain species with almost sessile carpels and closely reticulate leaves that grows above 4,000 feet in the montane forest. The phytochemical and biological properties of *P*. *Monticola* have not been extensively studies, although methanolic extract of bark has been reported to contain oxoaporphine alkaloids and phenolic acids with potential anti-diabetic effects in rats with Type 2 diabetes [10]. In the present study, two benzopyran derivatives, namely (6*E*,10*E*) isopolycerasoidol (**1**) and a new derivative, (6*E*,10*E*) isopolycerasoidol methyl ester (**2**) were identified and isolated from methanol extracts of *Pseuduvaria monticola* leaves. (**1**) was first isolated and phytochemically reported from *Polyalthia sclerophylla* in the same family [11]. However, studies to examine the pharmacological activities of benzopyran derivatives are limited. Therefore, we investigated the anti-proliferative and cytotoxic effects of (**1**) and (**2**) using a variety of *in vitro* cell-based assays. We show that (**1**) and (**2**) induced mitochondrial-mediated apoptosis in human breast cancer cell lines, which provides the first pharmacological evidence for their future development as anticancer agents.

## Materials and Methods

### General experimental procedures

^1^H- and ^13^C-NMR spectra were obtained on a JEOL ECX 500 MHz (Japan). HR-ESI-MS spectra were analysed on a LCMS-QTOF (AB Sciex, USA) using a C18 column (Waters Xbridge, 2.2 × 50 mm, 2.5 μm) at 40 ^o^C at a flow rate of 0.5 mL/min. UV spectra were recorded on a Shimadzu UV-250. IR spectra were recorded on a Perkin Elmer 1600. All solvents used were of AR and HPLC grade. Water was purified using a Milli-Q purification system (Millipore Corp, Bedford, USA).

### Plant material

*P*. *monticola* was collected from the montane forest located at Cameron Highlands, Pahang, Malaysia in October, 2011. No specific permission was required for the collection of this plant because it is a common local plant and the forest is accessible to public. This study did not involve endangered or protected species. The plants were identified by Mr Teo Leong Eng from the Department of Chemistry, Faculty of Science, University of Malaya. Voucher specimen (HIR 0009) was deposited in the herbarium of the Chemistry Department, University of Malaya.

### Extraction, isolation and HPLC analysis

**Fig 1:**
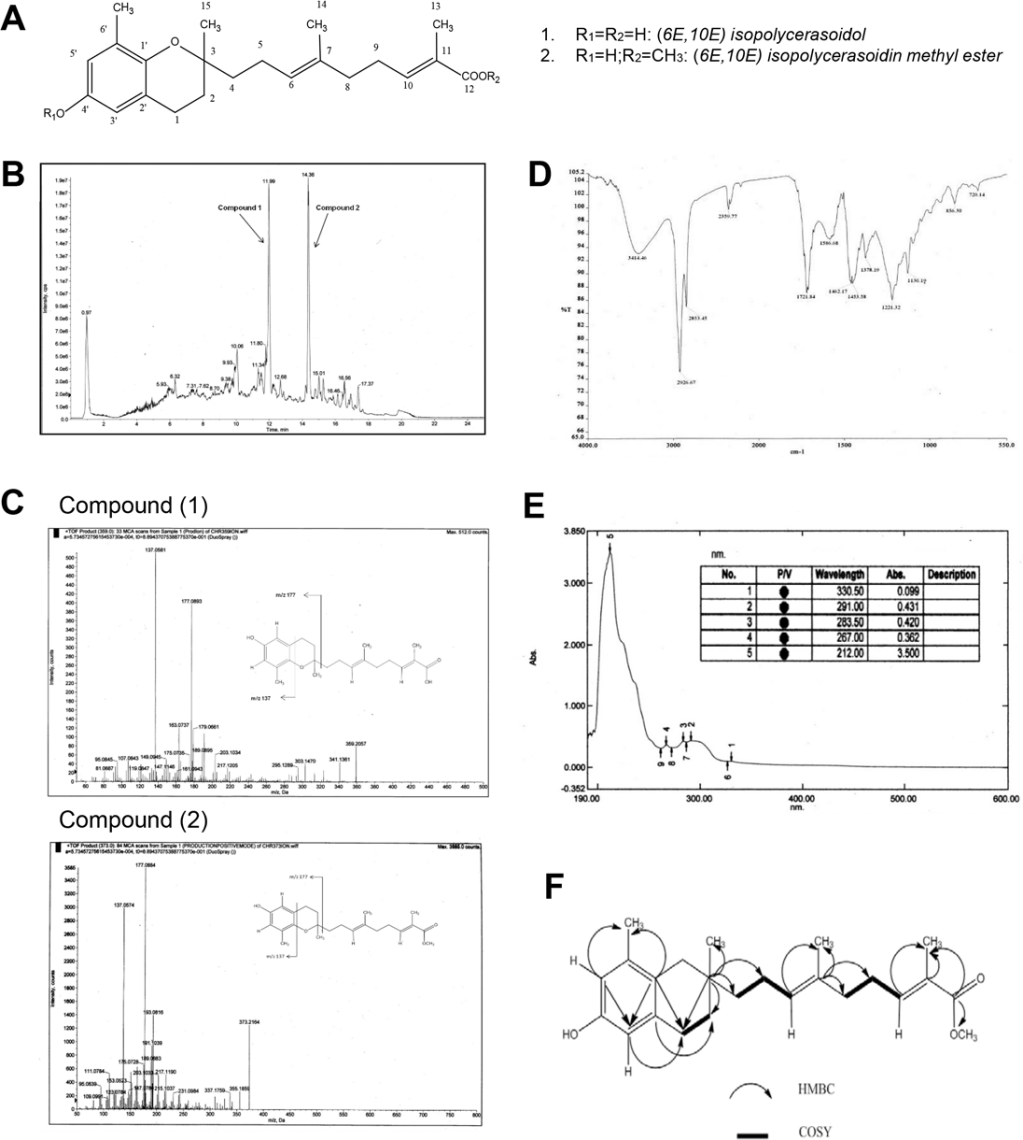
Phytochemical profile of compound (1) and (2). (A) Chemical structure of compound (**1**) and (**2**). (B). LCMS-QTOF total-ion-chromatogram (TIC) of (**1)** and (**2**) in methanolic leaf extract. (C) Product ions of (**1)** and (**2)** showing fragment ions at m/z 137 and m/z 177 by LCMS-QTOF. (D) COSY and HMBC correlation of compound (**2)**.

The dried and ground leaves of *Pseuduvaria monticola* (300 g) were first defatted with n-hexane for 24 h to remove the chlorophyll. The dried materials were then extracted with methanol (1 L) for three days at room temperature. The extract was then filtered and concentrated to dryness under reduced pressure to yield the methanol extract (25.7 g). Full scan total ion chromatogram (TIC) of the methanol extract showed two distinctive peaks which were then selected for isolation (Fig 1B). The methanol extract (300 mg) was subjected to a SPE clean-up procedure using SPE cartridges CEC 18 (UCT, PA, USA)) prior to fractionation by preparative HPLC (Gilson GX-281/322/156), using a reverse phased C18 column (Waters Nova-Pak, 25 × 100 mm, particle size 6 μm). The analyses were performed with a linear gradient from 0–100% acetonitrile in 80 min at a flow rate of 12 mL/min using acetonitrile in 0.1% formic acid (mobile phase B) and water in 0.1% formic acid (mobile phase A). HPLC chromatograms were monitored at 250–400 nm. TLC patterns from peak fractions were monitored and pooled together and analysed by LCMS-QTOF. The yields for compound (**1**) and (**2**) were 2.1 mg and 2.3 mg respectively.

**(6E,10E) isopolycerasoidol (1):**yellow oil; [α] ^25^_D—_11.11^o^ (*c* 0.009 MeOH); UV (MeOH) λ_max_ 211, 296 nm; IR (CHCl_3_) *v*_max_ 3357, 2927, 2853, 1693, 1470, 1378, 1221 cm^-1^; ^1^H and ^13^C NMR data; HRESITOFMS (High Resolution Electrospray Ionization Time of Flight Mass Spectrometry) m/z 359.2057 [M+H]^+^ (calcd for C_22_H_30_O_4,_ 359.4000).

**(6E, 10E) isopolycerasoidol methyl ester (2):**yellow oil; [α] ^25^_D_ - 12.5^o^ (*c* 0.008 MeOH); UV (MeOH) λ_max_ 212, 267 nm; IR (CHCl_3_) *v*_max_ 3414, 2926, 2854, 1721, 1462, 1378, 1221 cm^-1^; ^1^H and ^13^C NMR data; HRESITOFMS m/z 373.2164 [M+H]^+^ (calcd for C_23_H_32_O_4,_ 373.4220).

### Cell culture

Human breast cancer cell-lines, (MCF-7 and MDA-MB-231) and human breast normal epithelial cell-line (MCF-10A) were purchased from American Type Culture Collection (ATCC, Manassas, VA). Cells were grown in Dulbecco’s Modified Eagle Medium (DMEM, Life Technologies, Inc, Rockville, MD) supplemented with 10% heat-inactivated fetal bovine serum (FBS, Sigma-Aldrich, St. Louis, MO), 1% penicillin and streptomycin. Details of the senescent cancer-associated fibroblast strain, BICR78F, (a generous gift from Professor EK Parkinson) have been documented previously [12, 13]. The cells were cultured DMEM supplemented with 10% (v/v) fetal bovine serum (FBS) and 2 mM L-glutamine. Cells were cultured at 37°C in a humidified atmosphere with 5% CO_2_ and cells in exponential growth phase (approximately 70–80% confluence) were used in experiments.

### MTT cell viability assay

The cytotoxic effects on cancer cells were determined using MTT assays. 1.0 × 10^4^ cells were seeded into 96-well plates and incubated overnight at 37 °C in 5% CO_2_. After 24 h, the cells were treated with (**1**) or (**2**) using a two-fold dilution series of six concentrations (200 μM to 6.25 μM) or DMSO alone (negative controls) and further incubated for 48 h. MTT solution (3-(4,5-dimethylthiazolyl-2)-2,5-diphenyltetrazolium bromide; 2 mg/ml final concentration) was added and after 2 h the formazan crystal was dissolved in DMSO. The absorbance of the wells at 570 nm was determined using a Tecan M1000 Pro microplate reader (Männedorf, Switzerland). The percentage of viable cells was calculated as the ratio of the absorbance of compound-treated cells to the absorbance of DMSO-treated control cells [14]. IC_50_ was defined as the concentration of the extract that cause a 50% percent reduction of the absorbance of treated cells compared to DMSO-treated control cells. The experiments were carried out in triplicate.

### Real-time cell confluency and morphological analysis

*In vitro* cytotoxic effects of compound-treated and untreated cells were surveyed using a live cell analyzer JuLi Br system (NanoEntek, Seoul, Korea). The live cell analyzer monitored cell confluency and morphology by recording cell images at 1-hour intervals. On the first day, 5 × 10^5^ cells were seeded in each flask. During log growth phase, cells were treated with 50 μM of (**1**), (**2**) or left untreated and monitored continuously for 24 h.

### Reactive Oxygen Species (ROS) assays

ROS assays were carried out as reported previously [15]. Briefly, 1 × 10^4^ cells per well were seeded in 96-well plates. Cells were treated with (**1**) or (**2**) at the indicated concentrations for 24 h. Dihydroethidium (DHE) dye was added into live cultures for 30 min. Cells were then fixed and washed with wash buffer, following the manufacturer’s instructions. Stained cells were visualized and images acquired using a Cellomics ArrayScan HCS reader (Thermo Scientific). Fluorescence intensities of DHE dye in the nucleus were quantified using the target activation bioapplication module.

### Multiparameter cytotoxicity and phospho p38 MAPK assays

A Cellomics multiparameter cytotoxicity 3 kit (Thermo Scientific) was used as described previously [16]. 1 × 10^4^ cells per well were plated in 96-well plates and incubated overnight. Compound (**1**) and (**2**) were added and further incubated for 24 h. Mitochondrial membrane potential (MMP) dye (excitation 552/emission 576) and the cell permeability dye (excitation 491/emission 509) were added to live cells and incubated for 1 h. Cells were fixed and stained according to the manufacturer’s instructions. For phospho p38 MAPK detection, mouse monoclonal anti-human phospho p38 MAPK (Thermo Scientific) was added to the fixed cells for 1 hour. Cells were washed three times with PBS before adding Alexa fluor 488 secondary goat anti mouse antibody (Life Technologies, CA). Nuclei were stained with Hoechst 33258. Stained cells were visualized and images were captured using Cellomics ArrayScan HCS reader (Thermo Scientific).

### Flow cytometry

Apoptosis-mediated cell death was examined by a double staining method, using a FITC-labeled Annexin V/propidium iodide (PI) apoptosis detection kit (BD Bioscience, San Jose, CA), as previously described [14]. Briefly, cells were treated for 12 h and then harvested, washed in cold phosphate-buffered saline (PBS) twice and then stained with fluoresceinisothiocyanate (FITC)-conjugated Annexin V and PI dyes. The externalization of phoshotidylserine and the permeability to PI were evaluated using a FACS Calibur flowcytometer (BD Bioscience). Data from 10,000 gated events per sample were collected. Cells in early stages of apoptosis were positively stained with Annexin V, whereas cells in late apoptosis were positively stained with both Annexin V and PI.

### Caspase 3/7, 8, 9 bioilluminescent assays

Caspase 3/7 and 9 activities were measured using Caspase-Glo 3/7, 8 and 9 assay kits (Promega, Madison, WI), according to the manufacturer’s instructions. Briefly, cells were cultured in white-wall, optical bottom 96-well plates and treated with 25–100 μM **(1)** or **(2)**. At the indicated times, an equal volume of the assay reagent was added and the cells incubated for additional 1 h. The contents were mixed gently using a plate shaker at 300 to 500 rpm for 30 seconds and luminescence measured using a plate reader (Tecan M1000 Pro). Blank values were subtracted from experimental values and the experiments were carried out in triplicate.

### Quantitative real-time PCR (QPCR)

Total RNA was extracted using a RNeasy Mini Kit (Qiagen, UK) and subjected to reverse transcription using oligo(dT) primer and Superscript III (Invitrogen, USA). Q-PCR was performed in triplicate using the ABI Prism 7000 Sequence Detection System and TaqMan Gene Expression Assays. The assays were BCL2, Hs00608023_m1; Bax, Hs00180269_m1; Bim (Bcl2L11), Hs00708019_s1; Bad, Hs001889930_m1; Bid, Hs00609632_m; BCL2L1, Hs00236329_m1; Bak, Hs00832876_g1; MCL1, Hs01050896_m1 (Applied Biosystems, USA). GAPDH was amplified in the same reaction to serve as an internal control for normalization. Fold changes in gene expression were measured using the comparative threshold cycle method (ΔΔ Ct).

### *In situ* staining for β-galactosidase activity

Senescence detection was performed using a Senescence Detection Kit (Biovision), according to the manufacturer’s protocol. Briefly, 2 × 10^4^ cells were seeded into 24 well-plates. Cells were treated with compounds for 24 hours before washed in PBS (pH 7.4), fixed with 0.5 mL of fixative solution and incubated overnight at 37 C in freshly prepared staining solution mix containing X-gal (5-bromo-4-chloro-3-indolyl β-D-galactoside), citrate-buffered saline, pH 4.5. At the end of the incubation, cells were washed with PBS and examined at ×200 magnification for blue color development.

### Statistical analysis

All experiments were performed at least three times independently. The results were presented as mean ± standard deviation (SD). Statistical data were evaluated using an unpaired two-tailed Student’s *t-*test and were considered significant if P<0.05 or P<0.01.

## Results

### Characterization of compound (1) and (2)

A known prenylated benzopyran derivative, (6*E*,10*E*) isopolycerasoidol (**1**) and a novel (6*E*,10*E*) isopolycerasoidol methyl ester (**2**) were isolated for the first time from a methanol extract of *P*. *monticola* leaves using preparative HPLC purification (Fig 1A and 1B). The isolated structures were confirmed by means of UV, IR, 1D and 2D NMR and LCMS-QTOF spectroscopy methods.

Compound (**1**) was isolated as yellow oil and showed [α]_D_^25^ MeOH—11.11^o^ (*c* 0.009). The HRESITOFMS spectrum (Fig 1C) displayed a molecular ion peak [M+H]^+^ at m/z 359.2057 corresponding to the molecular formula of C_22_H_30_O_4_ (calcd 359.4000).The IR spectrum showed the absorptions bands for hydroxyl group (3357 cm^-1^), aliphatic C-H stretch (2927 cm^-1^), carbonyl group (1693 cm^-1)^ and aromatic ring function (1470 cm^-1^).The UV absorptions at λ_max_ 296 nm suggest the presence of C = C and peak at λ_max_ 211 nm suggest a conjugated system consistent with an aromatic chromopore [8]. The ^1^H-NMR (Table 1) spectrum showed four methyl signals at δ_H_ 1.25, 1.58, 1.89, 2.12 and two olefinic methines at δ_H_ 5.14 and 6.05. Two aromatic proton signals in meta position appeared at δ 6.34 (2H, *J* = 2.5 Hz, H-3') and δ 6.44 (2H, *J* = 2.5 Hz, H-5’) indicative of an AB system, which was apparent from their coupling constant. Resonances at δ 2.69 (2H, *m*) and δ 1.74 (2H, *m*) of an ABX_2_ system along with a methyl singlet at δ 2.12 (3H) and another methyl singlet at δ 1.25 (3H) attached to a carbon-bearing oxygen completed the substituted chromane moiety. The ^13^C-NMR revealed a total of 22 carbon signals attributed to four methyls δ 15.8 (C-14), 16.1 (C-6'), 20.6 (C-13), 24.0 (C-15), six methylenes, four methines and eight quartenary carbons (comprising of six aromatic carbon signals at δ 112.7 (C-3'), 115.7 (C-5'), 121.2 (C-2'), 126.6 (C-6'), 145.9 (C-1'),147.9 (C-4'), one oxygenated carbon signal at δ 76.8 (C-3) and two olefinic quartenary carbons at δ 127.4 (C-11) and δ 134.4 (C-7) (Table 1). The most downfield peak at δ _C_ 172.7 revealed the presence of carboxyl group. Based on the comparison of the ^1^H-, ^13^C-NMR, IR and MS spectral data with the known compound, the structure (**1**) was established as (6*E*,10*E*) isopolycerasoidol [11].

**Table 1:** ^1^H and ^13^C NMR data for compound (1) and (2), (CDCl_3_, δ in ppm, *J* in Hz) at 500 MHz.

Position	Compound (1) C_22_H_30_O_4_		Compound (2)C_23_H_32_O_4_	
	^**1**^ **H**	^**13**^ **C**	^**1**^ **H**	^**13**^ **C**
**1**	2.69 *m*	22.5	2.69 *m*	22.6
**2**	1.74 *m*	31.5	1.79 *m*	31.4
**3**	-	76.8	-	76.8
**4**	1.55–1.61 *m*	39.5	1.54–1.63 *m*	39.7
**5**	2.03–2.11 *m*	22.2	2.02–2.11 *m*	22.2
**6**	5.14 *t* (7.0,14)	125.1	5.16 *dt* (7.0,14)	125.1
**7**	-	134.4	-	134.4
**8**	2.04–2.10 *m*	39.1	2.04–2.10 *m*	39.2
**9**	2.56 *q* (7.5, 15)	28.2	2.55 *q* (7.0, 15)	28.1
**10**	6.05 *t* (7.0, 14)	146.1	5.92 *dt* (7.0, 14)	143.3
**11**	-	127.4	-	127.4
**12**	-	172.7	-	168.6
**13**	1.89 *s*	20.6	1.87 *s*	20.7
**14**	1.58 *s*	15.8	1.59 *s*	15.8
**15**	1.25 *s*	24.0	1.25 *s*	24.1
**1’**	-	145.9	-	145.9
**2’**	-	121.2	-	121.3
**3’**	6.34 *d* (2.5)	112.7	6.38 *d* (2.5)	112.7
**4’**	-	147.9	-	147.9
**5’**	6.44 *d* (2.5)	115.7	6.48 *d* (2.5)	115.7
**6’**	-	126.6	-	126.9
**CH3-6’**	2.12 *s*	16.1	2.12 *s*	16.2
**COOCH3-12**	-	-	3.72 *s*	51.3

Compound (**2**) was isolated as yellow oil and showed [α] _D_^25^ MeOH—12.5^o^ (*c* 0.008). The HRESITOFMS spectrum (Fig 1C; lower) revealed a molecular ion peak [M+H]^+^ at m/z 373.2164 corresponding to the molecular formula of C_23_H_32_O_4_ (calcd 373.4220). It was noted that the molecular formula of compound (**2**) was more by 15 amu than that of compound (**1**). The IR spectra revealed the presence of a hydroxyl group (3414 cm^-1^), an aliphatic C-H stretch (2926 cm^-1^), a conjugated carbonyl group (1721 cm^-1^) and an aromatic ring function (1462 cm ^-1^) similar to that of compound (**2**) (Fig 1D). The UV absorptions, λ_max_ at 291 and 212 nm confirmed the existence of a chromophore system in the compound (Fig 1E). The structure was further established by 1D and 2D NMR spectra. The ^1^H-NMR spectrum of (**2**) (Table 1) was similar to that of compound (**1**) except for an additional presence of one methoxy proton signal at δ 3.72 (3H) while its ^13^C-NMR spectrum showed a methoxy peak at δ _C_ 51.3 that could suggest a methoxyl group linked to the aromatic ring or a methoxy proton of the ester functionality (Table 1). An AB system can be deduced from the assignments of meta-coupled protons (δ 6.34 and 6.44, d, *J* = 2.5 Hz) indicating a substituted benzene ring A. The HMBC correlations between H-2 (δ_H_ 1.59, *m*), H-4 (δ_H_ 1.54–1.63, *m*), H-15(δ_H_ 1.25, *s*) and C-3 (δ_C_ 76.8) confirmed the isoprenyl side chain at C-3 linking to the benzopyran ring. The methyl esterification in the structure was further confirmed by HMBC correlations between H-OCH_3_ (δ_H_ 3.72, s), H-13 (1.87,s) and C-12 (δ_C_ 168.6) (Fig 1F). It can be observed that the carbonyl peak at δ_C_ 168.6 was slightly less deshielded compared to that of compound (**1**) (δ_C_ 172.7) due to the electron-donating effect of the methoxy group. The rest of HMBC correlations affirmed the benzopyran structure in the compound (Fig 1F). In the ^1^H-^1^H COSY spectrum (Fig 1F), the allyl protons at H-6 and H-10 display crosspeaks with H-4, H-5, H-8 and H-9 confirming a prenylated side chain in this molecule. Combined analysis of the ^13^C NMR (Table 1) showed 23 carbon signals consistent with four methyls δ_C_ 15.8, 16.2, 20.7, 24.1, six methylenes, four methines and eight quartenary carbons (comprising of six aromatic carbon signals at δ_C_ 112.7 (C-3'), 115.7 (C-5'), 121.2 (C-2'), 126.6 (C-6'), 145.9 (C-1'),147.9 (C-4'), one oxygenated carbon signal at δ 76.8 (C-3) and two olefinic quartenary carbons at δ_C_ 127.4 (C-11) and 134.4 (C-7), and one methoxy at δ_C_ 51.3 and this again showed similar carbon signal pattern of compound (**1**). MS/MS fragmentations provided more information on the structure. The main mass fragments were observed at m/z 137 (C_8_H_9_O_2_) and m/z 177 (C_11_H_13_O_2_) instead of diagnostic peaks at m/z 151 (C_9_H_11_O_2_) and m/z 191(C_12_H_15_O_2_) (Fig 1C; Lower). It can be deduced that the methoxy group is not attached to the aromatic ring. Therefore, based on DEPT,COSY, HMBC experiments and MS/MS fragment ions, compound (**2**) is proposed to be a methyl ester of (6*E*,10*E*) isopolycerasoidol, a new benzopyran derivative that has not been reported before. The complete assignments of ^1^H- and ^13^C- NMR are listed in Table 1.

### Compound (1) and (2) reduce cell viability and induce apoptosis but do not cause cellular senescence

The cytotoxic effects of (**1**) and (**2**) on human breast cancer cell-lines (MCF-7 and MDA-MB-231) and a human normal breast epithelial cell-line (MCF-10A) were evaluated using MTT assays. Results showed that relatively high cell viability inhibitory effect was observed in both breast cancer cell lines compared to normal breast cell-line after 48 h of treatment. The IC_50_ values are shown in Table 2.

**Table 2:** Cytotoxic effect of compound (1) and (2) on MCF-7 and MDA-MB-231 (human breast adenocarcinoma cell lines) and MCF-10A (human normal breast epithelial cells).

Cell-lines	IC_50_ (μM)	
	Compound (1)	Compound (2)
MCF-7	59 ± 5.1	43 ± 2.4
MDA-MB-231	76 ± 8.5	58 ± 2.6
MCF-10A	94 ± 5.9	90 ± 4.7

**Fig 2:**
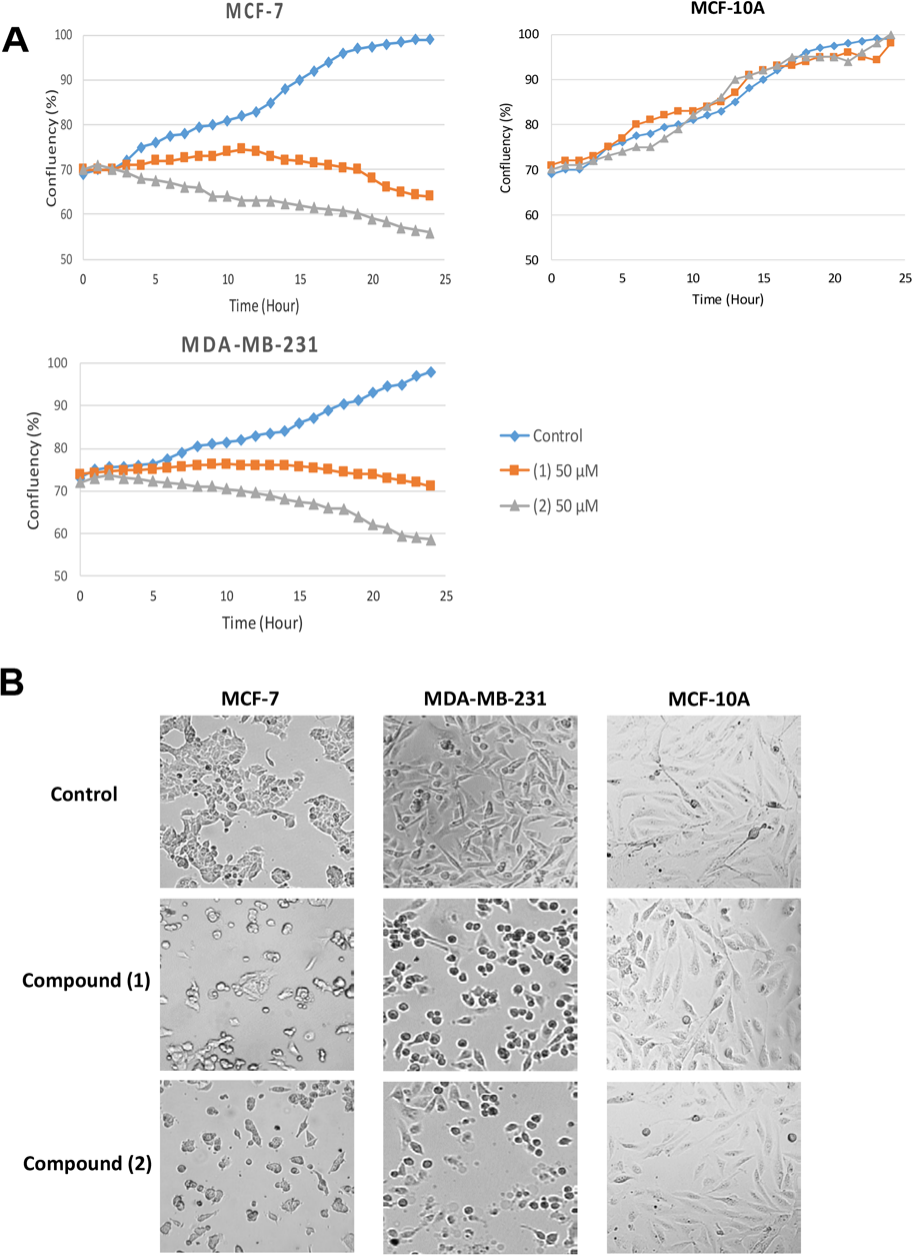
Effect of compound (1) and (2) on cell growth and cellular morphology. Cells were treated with 50 μM (**1**) or (**2**) or DMSO alone and images were captured using a live cell image analyzer JuliBr system for 24 hours. (A) Cell confluency was determined every hour. Addition of compound (**1**) or (**2**) lead to decreased cell confluency of both breast cancer cell-lines (MCF-7 or MDA-MB-231) and human normal breast epithelial cell-line (MCF-10A) compared to untreated control (B) Cell shrinkage and formation of apoptotic bodies were observed in (left) MCF-7 or (right) MDA-MB-231 cells after 24 hours treatment with **(1)** or **(2)**.

Next, real-time cell proliferation monitoring assays were carried out to monitor the growth pattern and morphological changes of MCF-7 or MDA-MB-231 breast cancer cells treated with (**1**) and (**2**). We observed that compound (**1**) or (**2**) caused a significant decreased in cell growth as reflected in the reduction of cell confluency after 24 h treatment (Fig 2A). In contrast, cell proliferation was not affected in MCF-10A treated with similar dosage. Cell shrinkage and apoptotic body formation were observable after 24 h of treatment in MCF-7 and MDA-MB-231, but not in MCF-10A (Fig 2B). Moreover, the number of attached cells in compound (**1**) or (**2**)-treated samples was significantly reduced compared to the untreated control (Fig 2B).

**Fig 3:**
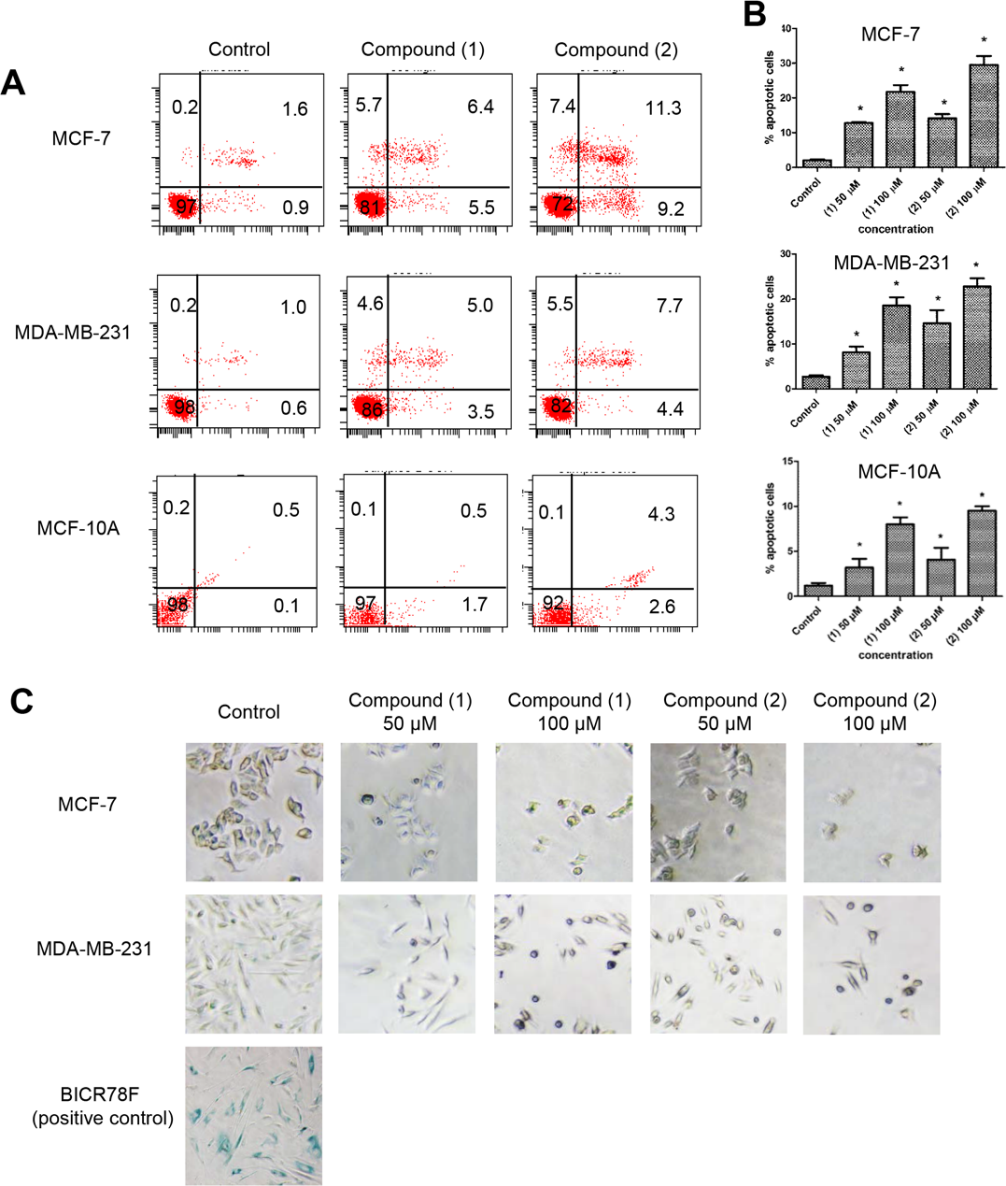
Compound (1) and (2) induce apoptosis rather than senescence in breast cancer cells. (A and B) MCF-7, MDA-MB-231 and MCF-10A cells were stained with Annexin V/PI and subjected to flow cytometry analysis. The four quadrants represent living cells (Annexin V-PI-), early apoptotic (Annexin V+PI-), late apoptosis (Annexin+PI+) or necrotic (Annexin V-PI+) stages. Values shown were percentages of each quadrant. *P<0.05, in comparison to control. (C) MCF-7 and MDA-MB-231 cells were treated with DMSO (solvent), (**1**) or (**2**) for 24 hours before subjected to *in situ* senescence-associated β-gal staining at pH 6. The development of blue color was examined by bright field microscopy. (Magnification 200X). Right panel: senescent cancer-associated fibroblast strain, BICR78F was included as a positive control for senescence-associated β-gal staining.

To confirm that reduced cell viability is due to induction of apoptosis in MCF-7 and MDA-MB-231 cells, we stained the cells with Annexin V/propidium iodide (PI) and performed flow cytometry analyses. Our result showed that Annexin V+/PI—apoptotic cell population increased in a dose-dependent manner (approximately 10% to 30%) in both MCF-7 and MDA-MB-231 cells treated with (**1**) or (**2**) compared to control at 12 h post treatment (Fig 3A and 3B). In contrast, MCF-10A was less affected by (**1**) or (**2**) treatment, with apoptotic cells constituting <10%.

To examine whether (**1**) or (**2**) could induce replicative senescence in cancer cells, treated MCF-7 and MDA-MB-231 cells were analyzed for senescence-associated β-gal activity *in situ* by incubation with X-gal at pH 6.0. As a positive control, we included a senescent cancer-associated fibroblast strain, BICR78F. As shown in Fig 3C, BICR78F cells were stained positively for senescence-associated β-gal activity (blue), whilst MCF-7 and MDA-MB-231 cells treated with (**1**) or (**2**) demonstrated very low β-galactosidase activity, indicating that neither compound caused cellular senescence.

### Compound (1) and (2) induce ROS generation

**Fig 4:**
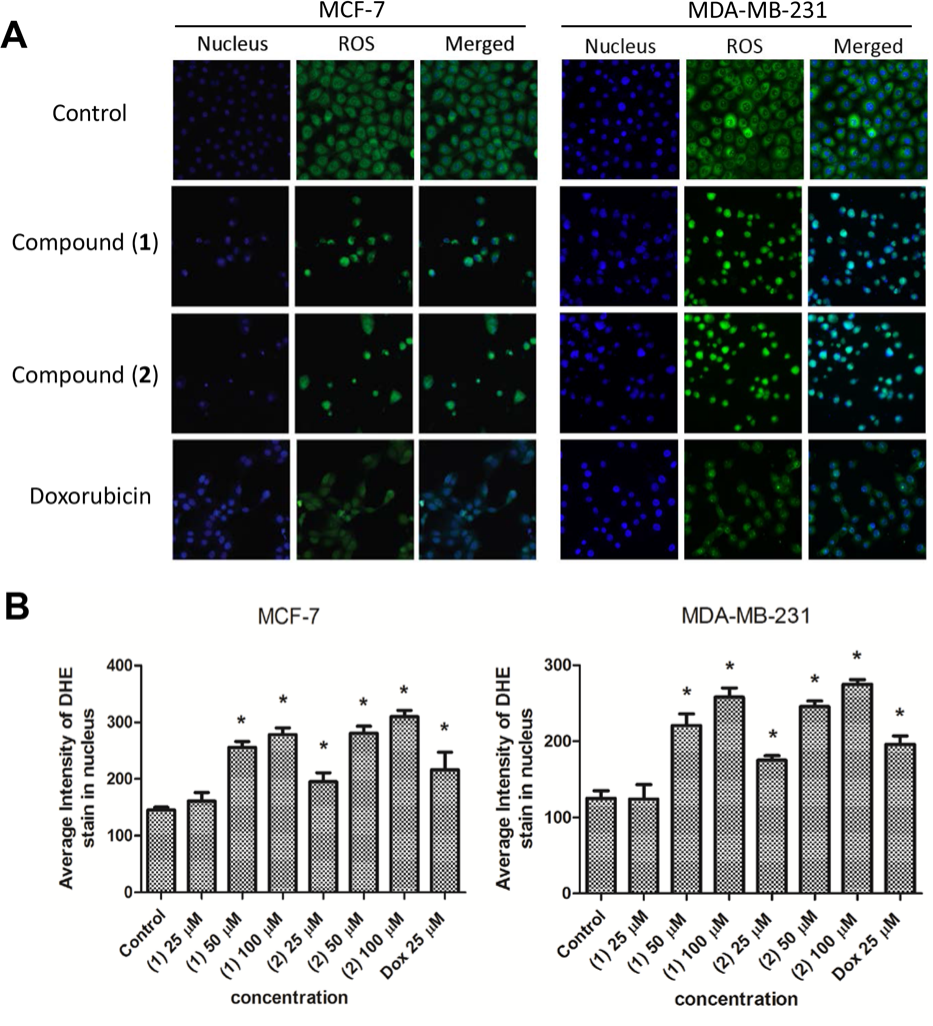
Compound (1) and (2) enhance ROS production in breast cancer cells. (A) Fluorescence images of (**1**) or (**2**)-treated MCF-7 or MDA-MB-231 cells stained with DHE dye for 30 mins to monitor ROS production. Doxorubixin (Dox) was used as a positive control. (B) Histogram showing the mean ± SD value of intensities for ROS as measured by a HCS system. *P<0.05, in comparison to control.

The presence of cytotoxic compounds could lead to oxidative stress, which is associated with increased production of oxidizing species or a significant decrease in the effectiveness of antioxidant defences in cells. Therefore, we measured ROS production in MCF-7 and MDA-MB-231 cells following treatment with (**1**) or (**2**) using DHE dye. Fluorescence increased markedly in the nuclei of compound (**1**) or (**2**)-treated cells compared to negative controls (Fig 4A and 4B), indicating that (**1**) and (**2**) induced the production of ROS in breast cancer cells.

### Effect of compound (1) and (2) on membrane permeability and MMP

**Fig 5:**
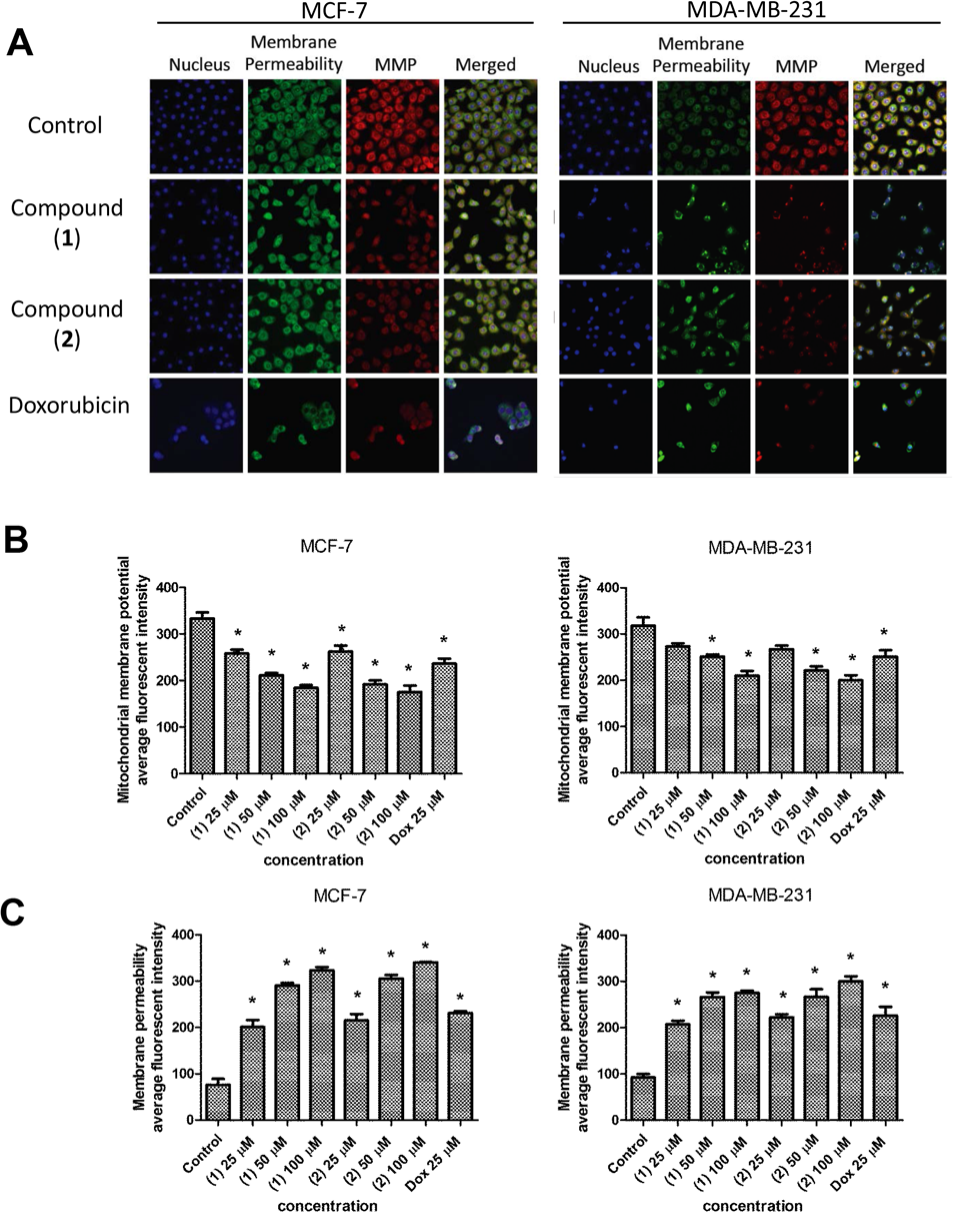
Effect of (1) and (2) on nuclear morphology, membrane permeabilization and MMP in MCF-7 and MDA-MB-231 cells. (A) Representative figures showing changes in DNA content (blue), cell permeability (green) and MMP (red) distribution patterns in control and treated cells. Doxorubixin (Dox) was used as a positive control. (B) Histogram showing the mean ± SD value of intensities cell permeability and MMP measured by a HCS system. *P<0.05, in comparison to control.

Next, we utilised a multiparameter cytotoxicity kit 3 to examine the effect of (**1**) and (**2**) on plasma membrane integrity, nuclear morphology and MMP. For this purpose, we stained MCF-7 and MDA-MB-231 cells with Hoechst 33342, membrane permeability dye and MMP. Nuclei of control cells were round, whilst nuclei of treated cells were condensed (Hoechst; Fig 5A). In addition, we observed a dose-dependent increase of membrane permeability and decreased MMP stain in both MCF-7 and MDA-MB-231 cells following treatment with (**1**) and (**2**) (Fig 5A and 5B).

### Compound (1) and (2) activate caspases 3/7 and 9

**Fig 6:**
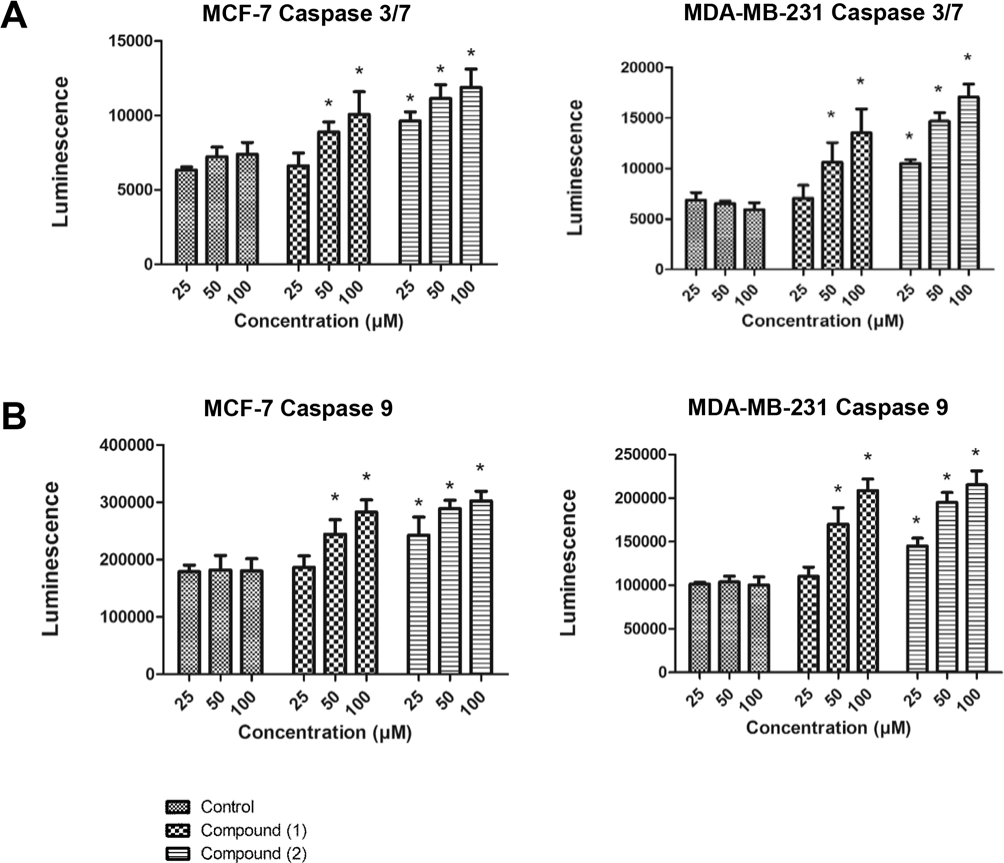
Effect of (1) or (2) on caspase activity. MCF-7 or MDA-MB-231 cells were treated with the indicated doses of compound (**1**) or (**2**) for 12 h prior to measuring the levels of (A) active caspase 3/7 and (B) active caspase 9 activities. The bars represent the mean ± SD. *P<0.05, in comparison to control.

Caspases are present in the proforms (inactive) and become active after site-specific cleavage to participate in the process of apoptosis. To determine whether caspases were involved in apoptosis induction by (**1**) and (**2**), the levels of active caspases 3/7, 8 and 9 were examined. Treatment of MCF-7 and MDA-MB-231 cells with (**1**) and (**2**) for 12 h resulted in a dose-dependent increase in activated caspases 3/7 and 9 (Fig 6A and 6B). Caspase 8 was not activated by any of the compounds in either cell line (data not shown).

### Compound (1) and (2) down-regulate *BCL2* and enhance accumulation of phosphorylated p38 MAPK in the nucleus

**Fig 7:**
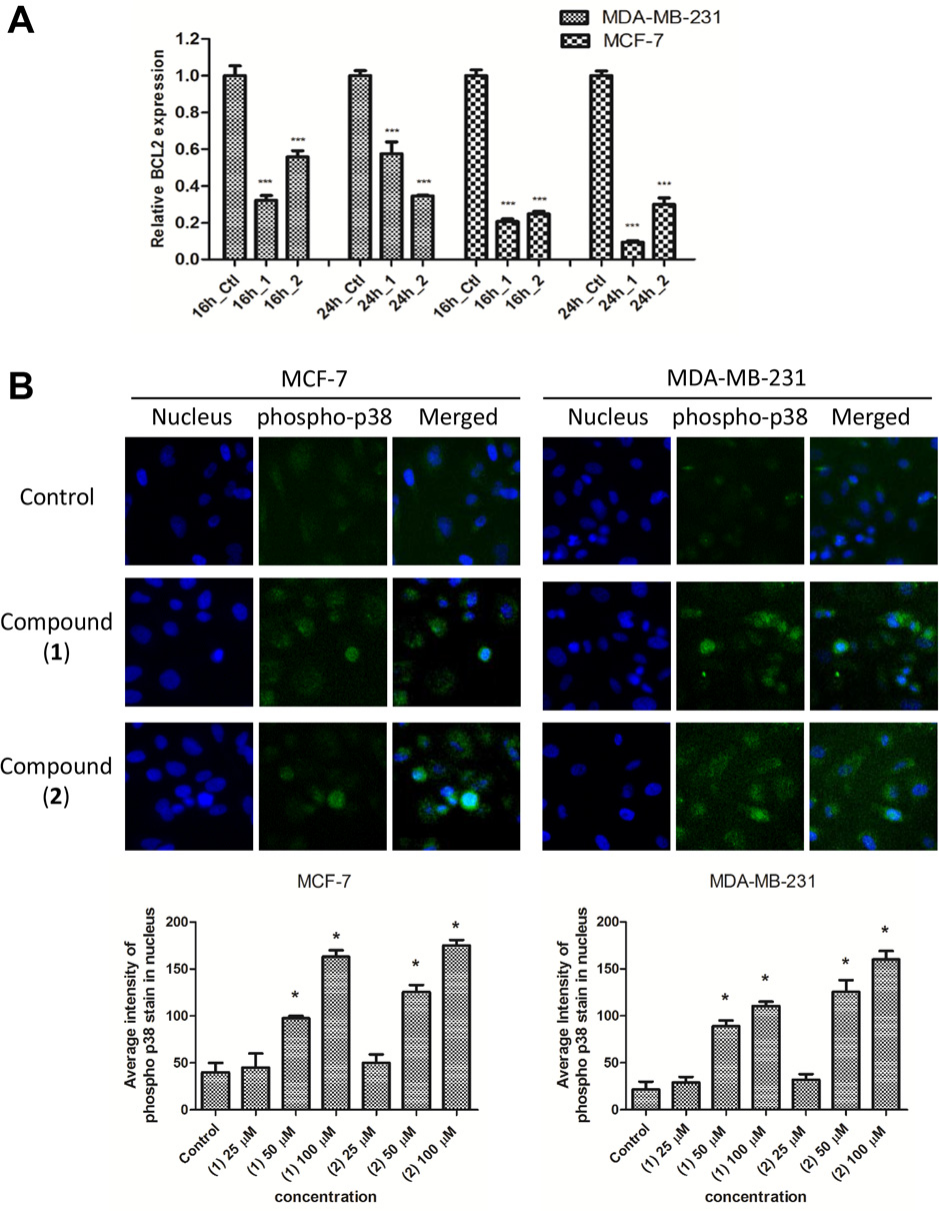
(1) and (2) modulate BCL2 and p38 MAPK signaling pathway. (A) QPCR analysis of *BCL2* expression in MCF-7 and MDA-MB-231 cells treated with IC_50_ concentration of (**1**) or (**2**) for 16 h or 24 h. The bars represent mean +/- SD and the control, untreated cells values were normalized to 1. GAPDH served as an internal control for normalization. Data were representative of at least three independent experiments. ***P<0.01, in comparison to control. (B) Representative figure showing nuclear accumulation of phospho p38 MAPK (green) in the nucleus (blue) of MCF-7 or MDA-MB-231 cells treated with (**1**) or (**2**) for 24 h. The fluorescent intensity of phospho p38 MAPK in the nucleus was quantitated and presented in bar charts. *P<0.05, in comparison to control.

Collectively, our data demonstrate that (**1**) and (**2**) induce apoptosis, increase membrane permeability, decrease MMP and activate caspases involved in the intrinsic apoptosis pathway in breast cancer cells, results which suggest the possible involvement of the BCL2 family of proteins. Compound (**1**) and (**2**) resulted in a significant reduction of *BCL2* mRNA levels in MCF-7 and MDA-MB-231 cells at 16 h and 24 h post treatment (Fig 7A). The expression of other *BCL2* members such as *BAD*, *BAX*, *MCL1*, *and BCL2L11* in compound (**1**) and (**2**)-treated cells was either heterogeneous or gave no mechanistic insights into the observed apoptosis induced by (**1**) and (**2**) (data not shown).

Since p38 MAPK, is a redox sensitive kinase which could be activated in response to oxidative stress, we examined the level of phosphorylated p38 MAPK in both breast cancer cell-lines treated with compound (**1**) or (**2**). In resting state, p38 MAPK is not phosphorylated, whereas activation of p38 MAPK translocates this molecule from cytoplasm to the nucleus. As shown in Fig 7B, we observed a dose-dependent accumulation of phosphorylated p38 MAPK in the nucleus of MCF-7 and MDA-MB-231 treated with (**1**) or (**2**), compared to controls. Together, our data indicate that compound (**1**) and (**2**) can modulate both BCL2 and p38 MAPK signaling pathways in breast cancer cells.

## Discussion

Screening natural products for anticancer properties has the potential to identify compounds which offer greater efficacy and less toxicity than synthetic drugs. Studies of the phytochemical properties of *P*. *monticola* are limited compared to other *Pseuduvaria species* [17]. In the present study, phytochemical investigation of a methanol extract of the leaves from *P*. *monticola* led to the isolation and structure elucidation of two prenylated benzopyran derivatives, (**1**) and a new compound, (**2**) based on various spectroscopic techniques. Of note, compound (**2**) is not an artifact from the esterification of compound (**1**) as it was also detected in a hexane extract based on GC/MS analysis (data not shown). The present study is the first to report the anti-proliferative effects of the isolated compounds, (**1**) and (**2**), on cancer cells. The anticancer effects could be attributed to the aromatic ring bearing the hydroxyl group for compound (**1**) and (**2**). The results showed that (**2**) is comparable to (**1**), indicating that the addition of a methoxy group in (**2**) did not affect the cytotoxic and pro-apoptotic activity of the compound.

We observed that the levels of ROS in (**1**) and (**2**) treated cells were significantly elevated. Excessive ROS production may cause a failure in the suppression of anti-apoptotic factors, and hence could lead the cells to apoptosis [18]. The BCL2 family of proteins control the apoptotic threshold of a cell and the ratio of anti-apoptotic and pro-apoptotic proteins within this family determines cell fate decisions [19, 20]. It has been known for some time that ROS-activated transcription factors can negatively regulate the transcription of *BCL2* [21]. We showed that treatment of both MCF-7 and MDA-MB-231 breast cancer cell lines with (**1**) and (**2**) results in the transcriptional down-regulation of *BCL2*. DNA damage by ROS is thought to lead to transcriptional activation of the tumour suppressor gene, p53, leading to the down-regulation of BCL2 and up-regulation of various pro-apoptotic genes [22]. Intriguingly, excessive generation of ROS may also be involved in the process of p38 activation. In the present study, (**1**) and (**2**) induced higher ROS levels in breast cancer cells, which may trigger the phosphorylation of p38 MAPK and lead to nuclear accumulation of p38 MAPK. Of note, p38 MAPK is a redox-sensitive kinase, which controls gene expression in response to oxidative stress. Although the exact mechanisms by which ROS can regulate p38 MAPK are not clear, a recent study showed that ROS can regulate p38 phosphorylation by inactivating MAPK phosphatases, leading to prolonged activation. Therefore, it seems likely that the elevated ROS levels induced by (**1**) and (**2**) in breast cancer cells leads to cell death by mechanisms that involve BCL2 and p38 MAPK.

Mitochondria play a principal role in the regulation of cell-survival and cell-death, as the main source of cellular adenosine triphosphate (ATP) and ROS [14]. Disruption of MMP is one of the initial intracellular events, which occurs after induction of apoptosis [23]. A previous study has demonstrated that prenylated benzopyran derivatives isolated from *Polyalthia cerasoides* (Annonaceae) could inhibit mitochondrial electron transfer chain [24]. Therefore, we evaluated MMP via the use of fluorescent probes to examine the influence of the induced ROS on the function of mitochondria. Both compounds induced depolarization of mitochondrial membranes. Loss of MMP may lead to excessive release of cytochrome *c* into the cytosol that can bind to apoptotic activating factor-1 and trigger the caspase cascade [25]. Our findings demonstrate the involvement of the mitochondrial pathway in the induction of apoptosis by (**1**) and (**2**) in MCF-7 and MDA-MB-231 cells and we observed that the plasma membrane was also affected, causing a significant elevation in membrane permeability. This could be due to higher ROS production, which is known to cause lipid peroxidation [26].

Intrinsic mitochondrial signaling can be triggered by non-receptor-mediated pathways [27]. For example, the release of cytochrome c following loss of MMP results in the activation of caspases and subsequent cell death. In the present study, (**1**) and (**2**) induced the activation of the apical caspase, caspase 9, and the activation of downstream effector caspases 3/7 in breast cancer cell lines, indicating that the induction of apoptosis by (**1**) and (**2**) is mediated via the intrinsic pathway. By contrast, caspase 8 activity was not activated, which most likely excludes the involvement of the extrinsic pathway. These conclusions are further supported by the observation that (**1**) and (**2**) caused a significant transcriptional down-regulation of the anti-apoptotic gene *BCL2*.

## Conclusion

In conclusion, our results showed that that compound (**1**) and (**2**) isolated from *P*. *monticola* are promising dietary phytochemicals with pro-apoptotic activity that could be further investigated as anticancer agents. Collectively, our data demonstrate that both (**1**) and (**2**) increase membrane permeability, decrease MMP and activate caspases involved in the intrinsic apoptosis pathway in breast cancer cells by modulating BCL2 and p38 MAPK signaling molecules.
